# ASO Author Reflections: Oncolactation Guidelines for Patients with Breast Cancer

**DOI:** 10.1245/s10434-025-17793-w

**Published:** 2025-07-14

**Authors:** Helen M. Johnson, Katrina B. Mitchell

**Affiliations:** 1 Department of Breast Surgical Oncology, The University of Texas MD Anderson Cancer Center, Houston, TX USA; 2 Surgical Oncology, Ridley-Tree Cancer Center, Santa Barbara, CA USA

## Past

Despite growing appreciation for the importance of oncofertility counseling for patients with cancer,^1^ oncolactation—the intersection of cancer care and breastfeeding—remains underrecognized as an essential component of multidisciplinary oncologic treatment. While breast surgeons are often assumed to be well-equipped to manage lactation-related issues, we receive limited education about the functional mammary gland in medical school, general surgery residency, and breast surgical oncology fellowship. Indeed, a recent survey of members of the American Society of Breast Surgeons highlighted that many breast surgeons report inadequate training in lactation and a strong interest in evidence-based guidelines.^2^

## Present

To address an unmet need, the American Society of Breast Surgeons developed evidence-based guidelines for oncolactation for patients with breast cancer.^3^ These include recommendations for breast cancer screening in pregnant or lactating patients, management of breast cancer diagnosed during pregnancy or lactation, and counseling for breast cancer survivors who desire childbearing and breastfeeding. All aspects of cancer treatment, including surgery, radiation, and systemic therapy, require modifications during pregnancy and breastfeeding, and each of these therapies can impact future lactation. If breastfeeding is contraindicated during treatment, patients should be provided with not only pharmacologic but also psychosocial support for weaning.

## Future

Because these are the first published guidelines for oncolactation and are specific to breast cancer, additional resources are needed to guide physicians in the management of breastfeeding patients with other types of cancer. Many oncologic medical therapies are contraindicated during lactation. Breastfeeding women undergoing oncologic surgery require unique perioperative support irrespective of the surgical site. Evidence-based recommendations are needed to assist oncology teams on how to appropriately modify care for lactating women and when weaning is medically indicated. Oncolactation counseling should be offered to all patients with cancer, similar to current standards for oncofertility counseling.

## References

[1] Irene SuH, Lacchetti C, Letourneau J, et al. Fertility preservation in people with cancer: ASCO Guideline Update. *J Clin Oncol*. 2025;43(12):1488–515. 40106739 10.1200/JCO-24-02782

[2] Johnson HM, Teshome M, Singh P, Mitchell KB. Lactation Education for Surgeons: American Society of Breast Surgeons (ASBrS) survey demonstrates strong member interest in expanded training. *Ann Surg Oncol*. 2023;30(10):6125–32. 37452168 10.1245/s10434-023-13882-w

[3] Johnson HM, Miller ME, Mitchell KB, et al. Oncolactation for patients with breast cancer: executive summary from the American Society of Breast Surgeons. *Ann Surg Oncol*. 2025. 10.1245/s10434-025-17742-7. 40627104 10.1245/s10434-025-17742-7 PMC12534219

